# Genomic Characterization of Antibiotic-Resistant *Campylobacterales* Isolated from Chilean Poultry Meat

**DOI:** 10.3390/antibiotics12050917

**Published:** 2023-05-16

**Authors:** Macarena Concha-Toloza, Mónica Lopez-Cantillo, Jose Arturo Molina-Mora, Luis Collado

**Affiliations:** 1 Instituto de Bioquímica y Microbiología, Facultad de Ciencias, Universidad Austral de Chile, Valdivia 5110566, Chile; 2 Centro de Investigación en Enfermedades Tropicales (CIET) & Facultad de Microbiología, Universidad de Costa Rica, San José 11501-2060, Costa Rica

**Keywords:** *Campylobacter*, *Arcobacter*, *Helicobacter*, poultry, resistance, whole-genome sequencing, MLST, Chile

## Abstract

Due to the lack of knowledge about *Campylobacterales* in the Chilean poultry industry, the objective of this research was to know the prevalence, resistance, and genotypes of *Campylobacter*, *Arcobacter* and *Helicobacter* in 382 samples of chicken meat purchased in Valdivia, Chile. The samples were analyzed using three isolation protocols. Resistance to four antibiotics was evaluated by phenotypic methods. Genomic analyses were performed on selected resistant strains to detect resistance determinants and their genotypes. A total of 59.2% of the samples were positive. *Arcobacter butzleri* (37.4%) was the most prevalent species, followed by *Campylobacter jejuni* (19.6%), *C. coli* (11.3%), *A. cryaerophilus* (3.7%) and *A. skirrowii* (1.3%). *Helicobacter pullorum* (14%) was detected by PCR in a subset of samples. *Campylobacter jejuni* was resistant to ciprofloxacin (37.3%) and tetracycline (20%), while *C. coli* and *A. butzleri* were resistant to ciprofloxacin (55.8% and 2.8%), erythromycin (16.3% and 0.7%) and tetracycline (4.7% and 2.8%), respectively. Molecular determinants were consistent with phenotypic resistance. The genotypes of *C. jejuni* (CC-21, CC-48, CC-49, CC-257, CC-353, CC-443, CC-446 and CC-658) and *C. coli* (CC-828) coincided with genotypes of Chilean clinical strains. These findings suggest that besides *C. jejuni* and *C. coli*, chicken meat could play a role in the transmission of other pathogenic and antibiotic-resistant *Campylobacterales.*

## 1. Introduction

The order *Campylobacterales* is widely known because of its pathogenic genera *Campylobacter*, *Helicobacter*, and to a lesser extent *Arcobacter*. However, other members are known to play ecologically important roles in diverse niches and environments [1].

*Campylobacter jejuni* and *C. coli* produce most of the human bacterial gastroenteritis cases worldwide, and *C. jejuni* is also associated with post-infection sequelae such as Guillain-Barre syndrome [2,3,4]. Meanwhile, *A. butzleri*, *A. cryaerophilus* and *A. skirrowii* have been associated with gastrointestinal and systemic diseases [5,6]. Regarding the genus *Helicobacter*, *H. pylori* is the most relevant species due to its association with gastritis, peptic ulcers, and gastric cancer [7]. However, in recent years entero-hepatic *Helicobacter* (EHH) species have become increasingly important as emerging pathogens and potential zoonotic agents due to their link to intestinal and hepatobiliary diseases [8].

*Campylobacter jejuni/coli* reservoirs include a wide variety of animals, such as farm animals, pets, and wildlife [2,9]. Zoonotic transmission can occur through the consumption of food of animal origin or by drinking contaminated water, but poultry meat has been attributed as their main route of transmission [2,10,11]. In the case of *Arcobacter* and *Helicobacter*, their transmission routes are not entirely clear, but there is growing evidence that it is like that of *Campylobacter* spp. [8,12].

The human disease caused by *Campylobacter* is generally self-limited, and antimicrobial treatment is recommended only in severe, persistent, or recurrent infections and in immunocompromised patients [13]. Macrolides and fluoroquinolones are the first line of treatment for gastrointestinal infections, and tetracyclines and aminoglycosides for systemic infections, whereas in *Arcobacter* the use of tetracyclines has been suggested [14].

In recent years, an increase in antimicrobial resistance has been observed in clinically relevant *Campylobacterales* isolated from both animal reservoirs and human samples worldwide [15,16,17]. Consequently, the World Health Organization (WHO) has classified *Campylobacter* spp. and *Helicobacter pylori* as high-priority bacteria for the study of new antimicrobials due to the high levels of fluoroquinolones and clarithromycin resistance reported, respectively [16].

In Chile, a noteworthy increase in resistance to ciprofloxacin (CIP) and tetracycline (TET) has been observed in clinical strains of *Campylobacter* spp. over the past two decades [17,18]. Likewise, resistance to CIP, TET, and Erythromycin (E) has been reported in isolates obtained from chicken and bovine meat [3,19]. Globally, it has been suggested that this increase in antimicrobial resistance is mainly due to the indiscriminate use of antibiotics in poultry and livestock production, to which is added the ability of these microorganisms to survive in these conditions and, therefore, endure over time, although there are also other potential resistance sources [20,21,22].

Based on the above data and the fact that the transmission of *Campylobacter* in the ecosystem is multidirectional, this microorganism represents a One Health challenge [23]. Unfortunately, this approach is still relatively early in Chile compared to other South American countries such as Brazil, and the study of *Campylobacter* epidemiology is little considered; therefore, its detection in various sources is limited and focused on certain research groups [18,24].

So far, most studies on resistance in the poultry industry have focused on *C. jejuni* and/or *C. coli*. However, considering that different *Campylobacterales* reside in the intestines of birds, it is necessary to assess how selective pressures affect them as a group. Bearing this in mind, and the fact that there is a lack of knowledge about *Campylobacterales* present in the Chilean poultry industry, this study aims to reveal the prevalence, antimicrobial resistance, and genetic diversity of *Campylobacterales* present in retailed chicken meat samples in Chile.

## 2. Results

### 2.1. Prevalence and Distribution of Campylobacterales

A total of 226 out of 382 (59.2%) chicken meat samples tested positive for *Campylobacterales* using microbiological culture. In most samples, only one species was isolated (45.8%) (Table 1), while 11.5% (44/382) of the samples were positive for both *Campylobacter* spp. and *Arcobacter* spp. The most prevalent species was *A. butzleri* (37.4%; 143/382), followed by *C. jejuni* (19.6%; 75/382), *C. coli* (11.3%; 43/382), *A. cryaerophilus* (3.7%; 14/382), and *A. skirrowii* (1.3%; 5/382). It was not possible to isolate *Helicobacter* spp. in any sample with the culture media used (Table 2). However, *Helicobacter pullorum* was detected (14%) by PCR in a subset of samples.

As shown in Table 2, *Arcobacter* spp. was able to grow in all isolation protocols, while *Campylobacter* spp. could only be recovered in Bolton broth and mCCDA plates incubated at 37 °C under microaerobic conditions (Protocol B).

### 2.2. Antimicrobial Resistance

Susceptibility to antimicrobials was assessed phenotypically in 280 isolates (118 *Campylobacter* spp. and 162 *Arcobacter* spp.). *Campylobacter jejuni* showed resistance to CIP (37.3%) and TET (20%), with 5.3% strains resistant to both antibiotics. *Campylobacter coli* was resistant to CIP (55.8%), E (16.3%), and TET (4.7%), in which 16.3% strains presented simultaneous resistance to CIP-E and 2.3% strain to CIP-TET. All *Campylobacter* isolates were susceptible to Gentamicin (GEN) (Table 3).

*Arcobacter butzleri* was the only species of the genus with antimicrobial resistance confirmed by MIC, with 2.8% to CIP, 0.7% to E, and 2.8% to TET. All *Arcobacter* isolates were susceptible to GEN (Table 3). However, there were discrepancies between the results of the disk diffusion and minimum inhibitory concentration methods, where the resistant isolates only coincided in 50%, 33.3% and 5.6% for CIP, E and TET, respectively.

### 2.3. Molecular Mechanisms of Antibiotic Resistance

**Figure 1:**
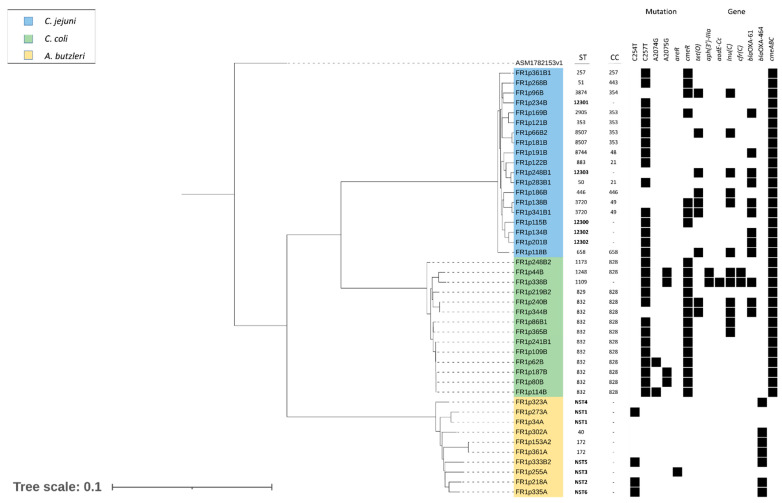
Phylogenetic trees based on whole-genome sequencing generated with Genome Blast Distance Phylogeny (GBDP). The presence or lack of molecular determinants of antimicrobial resistance of a subgroup of antibiotic-resistant *Campylobacterales* isolates is shown with the presence or absence, respectively, of a black block. The new STs are in bold type. NST: New Sequence. Type; Bar, 0.1.

Molecular antibiotic resistance determinants detected after whole-genome sequencing and functional annotation on selected samples are shown in Figure 1. All *Campylobacter* spp. strains resistant to CIP and TET had the C257T mutation in the quinolone resistance-determining regions (QRDR) of *gyrA* gene and the *tet(O)* gene, respectively. Meanwhile, the six *C. coli* strains resistant to E, presented the A2074G (2/6) or A2075G (4/6) mutation. The *erm(B)* gene, however, was detected in none of the strains. All *Campylobacter* strains presented the *cmeABC* gene. Moreover, 7 of 19 *C. jejuni* strains whose genome was sequenced and all *C. coli* had mutations in the *cmeR* gene. Additionally, resistance determinants were found for certain antibiotics that were not phenotypically tested. Some *C. jejuni* possessed the *lnu(C)* gene (6/19) and the presence of *bla*OXA-61 (9/19). While *C. coli* presented the gene *aph(3′)-III* (2/14), *aadE-Cc* (1/14), *cfr*(C) (2/14) and the presence of *bla*OXA-61 (3/14).

All *A. butzleri* strains that had resistance phenotype to CIP had the C254T mutation in the QRDR of *gyrA* gene. Moreover, in one *A. butzleri* strain resistant to E, we found a mutation in the *areR* gene, suggesting an overexpression of the AreABC pump. Interestingly, despite observing phenotypic resistance to TET, no known specific molecular determinant was found for this antibiotic. However, most (7/10) strains had *bla*OXA-464.

### 2.4. Virulence Genes

Although it was not one of the main objectives of the study, virulence determinants were investigated using whole-genome sequences. It was found that the main genes in *Campylobacter* are *cadF, jlpA, porA, pebA, racR, dnaJ, pldA, ciaB, ceuE, iamb,* and *flaC*. The *cdtABC* toxins genes were found almost exclusively in *C. jejuni*, while the genes associated with T6SS were found in *C. jejuni* and *C. coli*. (Figure S1). In *A. butzleri,* the *cadF, cj1349, mviN, ciaB, pldA* and *irgA,* genes were found in all the isolates, while only some strains have *hecA* (2/10), *hecB* (6/10) and *iroE* (6/10) genes (Figure S2).

### 2.5. Genotyping

As shown in Figure 1, the 19 *C. jejuni* genome sequences were classified into 16 different STs, four of which had not previously been reported (2 by novel allele sequences and 2 by novel combinations of preexisting alleles), which were submitted to the *Campylobacter jejuni/coli* PubMLST database. The remaining twelve STs were grouped into nine different clonal complexes (CC-21, CC-48, CC-49, CC-257, CC-353, CC-354, CC-443, CC-446 and CC-658). In addition, five different STs were identified out of 14 *C. coli*, with four of them grouped in CC-828, while ST-1109 could not be assigned to any CC.

In *A. butzleri*, eight different genotypes were identified. According to the analysis in the *Arcobacter* spp. PubMLST, six of which had not previously been reported (3 by novel allele sequences and 3 by novel combinations of preexisting alleles). The remaining two STs corresponded to the ST-40 and ST-172. However, new allele/new ST numbers could not be assigned to these *Arcobacter* genes/strains, respectively, due to the lack of a curator for this species in the database at the submission date (February 2023) (Keith Jolley, personal communication).

## 3. Discussion

Although the existence of *Campylobacter* and taxonomically related organisms in chicken meat samples is well known, most of the studies worldwide have been focusing only on *C. jejuni* and, to a lesser extent, on *C. coli,* with other *Campylobacterales* rarely considered, even when they coexist in the same reservoir and are subject to the same selective pressures, due to exposure to antibiotics in the poultry industry [25].

In the current study, more than half of the chicken meat samples for sale in the city of Valdivia, Chile, were positive for different *Campylobacterales.* In total, six species were detected: *C. jejuni, C. coli, A. butzleri, A. cryaerophilus, A. skirrowii* and *Helicobacter pullorum*. This contrasts with previous studies, where up to four species for this type of sample have been reported [15,26]. As far as we are aware, this is the first study demonstrating a higher diversity of *Campylobacterales* coexisting in samples of retail chicken meat.

### 3.1. Prevalence and Distribution of Campylobacterales

The prevalence of *Campylobacter* spp. in chicken meat samples was 29.8%, with *C. jejuni* present in 19.6% and *C. coli* in 11.3%, figures that fall within the ranges reported worldwide [25]. Unlike the large number of studies carried out on chicken meat in North America and the European Union (EU), studies carried out in South America are scarce, and as such unrepresentative. Moreover, they are concentrated in just a few countries, mainly Brazil, followed by Argentina and Chile [3,27,28].

Some studies on poultry meat in Chile show *Campylobacter* spp. has a similar prevalence to *Salmonella* spp. [3,29]. Despite this, unlike *Salmonella* the diagnosis of *Campylobacter* is not included in the Chilean sanitary regulations for food [18]. This may be because of their infrequent diagnosis in clinical laboratories, in contrast to what happens in high-income settings such as the United States, Iceland, the Netherlands and New Zealand, where the high prevalence obtained from both clinical samples and poultry meat led to recommendations to control *Campylobacter* in processing plants and imported products [18,30]. However, despite the scarcity of clinical laboratory diagnoses, a progressive increase in its detection as a cause of diarrhea was observed in Chilean children under five years of age between 2013 and 2017, as well as in adults with diarrhea [18,31].

Studies on emerging *Campylobacterales* are very limited in South America. Indeed, this is the first report of *Helicobacter* spp. in chicken meat in this setting. Within the emerging *Campylobacterales, Arcobacter* spp. presented a prevalence of 40.8%, where the most isolated species was *A. butzleri* (37.4%)*,* predominating even over the genus *Campylobacter* spp. (Table 2) [19,32,33]. This could be due to the capacity of *A. butzleri* to grow in a wide range of temperatures, in different atmospheres and in aquatic environments. As such, it may be better able to survive in poultry industry facilities and be found in high concentrations in this type of food [32,34]. *Arcobacter butzleri* has been identified as an emerging foodborne pathogen and has been reported from Chilean patients with gastroenteritis [35,36]. However, like other emerging pathogens, there are no regulations on its detection in food, and its diagnosis in clinical samples is not done routinely [6,37].

It was not possible to isolate *Helicobacter* spp. This could be because *Helicobacter* spp. was present in low concentrations and/or in a viable but non-culturable state or because the protocol used was not ideal for isolating it from chicken meat, even though the media used have allowed the isolation of several EHH species in other biological samples [8,38,39]. However, a frequency of 14% for *H. pullorum* was obtained by molecular detection. This figure is close to the range reported in chicken meat in Iran (16–49%) [40,41]. There is only one previous record of this species from the bile of a patient with chronic cholecystitis in Chile [42]. As such, this is the first report of *H. pullorum* in Chilean chicken meat.

### 3.2. Antimicrobial Resistance and Virulence

There is a need to explain the increase in antibiotic resistance observed in clinical *Campylobacter* spp. strains over the last two decades in Chile [17,18]. While it has been attributed to the use of antimicrobials in the poultry industry, its cause is not known with certainty [18].

The results obtained show that the pattern of antibiotic resistance in *Campylobacter* spp. analyzed coincides with what has been reported in chicken meat and clinical strains worldwide [3,43]. According to phenotypic and genomic analyses, all *Campylobacter* strains resistant to CIP presented the C257T mutation in *gyrA* gene. This mutation is also associated with a gain in the fitness of *Campylobacter*, which would imply that strains with these mutations persist over time, even when the antibiotic is discontinued [44,45]. On the other hand, *Campylobacter* resistance to TET was similar to what had previously been reported for clinical strains, chicken meat and bovine liver in Chile [3,19]. All strains phenotypically resistant to TET possess the *tet(O)* gene in their genome [46]. Among the *Campylobacter* species, only *C. coli* presented resistance to E, which was associated with the presence of the A2074G and A2075G mutations in the 23S rRNA. These mutations generate a cost in the fitness of *C. jejuni,* but not in *C. coli*, which could explain the results obtained in this study [47,48]. The *erm (B)* gene was not detected in the genomes analyzed, though it has already been reported in strain from chicken skin samples from Peru and China [49,50].

All *Campylobacter* genomes contained *cmeABC* genes, which generate an efflux system or pump that contributes to *Campylobacter* intrinsic resistance to various antimicrobials, including CIP, E, and TET [51,52]. This pump has already been reported in Chile in *Campylobacter* spp. strains of bovine origin, as well as in clinical strains [19,53]. However, seven of the 19 *C. jejuni* genomes and all of *C. coli* (n = 14) presented mutations in the *cmeR* gene, which is associated with a greater expression of this multidrug efflux pump. Despite this, a difference in MICs was not observed between those that did and did not present the mutation [54]. To our knowledge, this is the first time that mutations in the *cmeR* gene is reported in Chilean strains.

Additionally, genomic analysis allowed us to detect antibiotic-resistance genes that were not phenotypically tested here, such as *lnu(C), cfr(C)* and *bla*OXA-61 [55,56]. Meanwhile, only the *C. coli* genomes presented molecular determinants of resistance to aminoglycosides (*aph(3′)-III* and *aadE-Cc*). Even though both are related to resistance to aminoglycosides, none of them confers resistance to gentamicin, in accordance with the phenotypic results obtained in this study [57].

Genomic analysis of resistance determinants has the potential to accurately predict resistance phenotypes [57]. As such, 27.3% of these *Campylobacter* spp. strains could be classified as multidrug-resistant (MDR), that is, strains with resistance to three or more classes of antibiotics, which coincides with the MDR reported in clinical samples in Chile [53]. This figure is lower than that obtained in other countries such as India (54.4%) and China (93.7%), in which a considerable increase in strains resistant to antibiotics has been observed over the years [58,59]. However, it is similar to other South American countries, like Brazil (13%) [60].

Similar phenotypic patterns of antimicrobial resistance reported in *Campylobacter* were observed for *A. butzleri*. The percentage of strains resistant to TET is within the range reported worldwide, while lower values were obtained for CIP and E [61]. A discrepancy was observed between the resistance results by DD and MIC in *Arcobacter* spp., mainly to TET. Since *Arcobacter* spp. does not have cut-off values in CLSI or EUCAST, most studies use the criteria and breakpoints listed for *C. jejuni/C. coli* in CLSI M45 [33,62]. However, the data obtained in this, and previous studies would suggest that *Campylobacter* cut-off values are not reliable for this microorganism and confirm the need for having own and standardized cut-off values for a trustworthy interpretation [63].

All the sequenced *A. butzleri* strains resistant to CIP carried the C254T mutation in the *gyrA* gene [63]. Additionally, all of them presented the *adeF* gene (with a low percentage of identity), associated with resistance to fluoroquinolones and tetracyclines [64]. A total of 70% of the *A. butzleri* genomes presented the previously reported *bla*OXA-464 gene, which is associated beta-lactams resistance [63,65]. Moreover, for the first time we report a mutation in the *areR* gene, involving an overexpression of the AreABC pump, which would correlate with the resistance to E observed in a strain, which could be supported with further gene expression analysis [66,67]. Meanwhile, the presence of the *tet(O), tet(A)* or *tet(W)*, which generate resistance to TET, were not found in these strains. As we currently have limited knowledge of molecular determinants of resistance in *Arcobacter*, the presence of an unknown gene conferring phenotypic resistance cannot be ruled out, which highlights the need to investigate them, more so given that this is a naturally transformable bacterium, and therefore, could acquire an antibiotic resistance gene by horizontal transfer [68].

Even though the same antimicrobials used in human medicine are not used in the poultry industry [18,69], those applied in avian production belong to the same families, as in the case of enrofloxacin, tylosin, and oxytetracycline, which are antibiotics belonging to the fluoroquinolones, macrolides and tetracyclines families, respectively, and authorized for use in poultry according to the Chilean registry of veterinary medicines [69]. It is important to know about the local use of these antibiotics as it has previously been shown that the use of enrofloxacin for the treatment of broiler chickens, generated resistance to CIP in 100% *C. jejuni*, obtaining a MIC ≥ 32 µg/mL, which persists after the end of treatment [70]. In the case of tylosin, its use in therapeutic concentrations has generated resistance to E in 33.3% of *C. coli* and 7.9% of *C. jejuni*, a situation that in some cases generates highly resistant mutants (MIC > 512 µg/mL) [71,72]. Significantly higher resistance to TET, meanwhile, was seen when broilers were treated with oxytetracycline, and this increased resistance to TET has even been reported in *Salmonella* spp. isolated from chicken meat in Chile, with a range of 95.4–100% resistance to this antibiotic [29,73,74].

On the other hand, although it was not one of the main objectives of the study, virulence determinants were also detected. In *Campylobacter* spp., the virulence genes found are consistent with those previously reported in Chile in different types of samples, where those associated with adhesion and invasion predominate. It was also found that *C. jejuni* presents a greater number of virulent genes than *C. coli* [3,53]. We have also reported here on virulence genes detected in the genomes of *A. butzleri*, which is an emerging pathogen that presents several virulence determinants homologous to genes present in *Campylobacter* spp. (Figure S2).

### 3.3. Epidemiology

At the time of writing, the genotypes of only 145 Chilean strains of *Campylobacter* spp. were available in the PubMLST database, which were obtained mainly from clinical samples [17,53]. This is around 200 and 300 times less in comparison with the USA and the United Kingdom, respectively, where the epidemiology of *Campylobacter* spp. has been extensively investigated. However, it is similar to the amount of data from South American countries such as Brazil and Ecuador.

This is the first Chilean study reporting *Campylobacter* MLST genotypes of chicken meat strains. The *C. jejuni* strains were grouped into clonal complexes (CC) previously reported in clinical strains isolated in Valdivia (CC-21, CC-48, CC-257 and CC-353) and Santiago (CC-21, CC-48, CC-49, CC-257, CC-353, CC-443, CC-446 and CC-658) [17,53]. One poultry isolate corresponded to ST-3874 belonging to CC-354, which has been previously reported in a bovine liver isolate in the same geographical area (Valdivia city) and has also been isolated from clinical samples in the USA and Canada [19,75]. A total of 21% of the *C. jejuni* strains (4/19) clustered in CC-353, being the most prevalent CC, a genotype known to cause human infections and colonize broiler products, as well as its association with resistance to quinolones [76,77,78].

*Campylobacter coli* presents a particular genetic population structure, in which at least three main clades have been described to date [79]. In this study, all the *C. coli* strains belonged to clade 1 (Figure S3), that are commonly isolated from farm animals and human gastroenteritis cases, unlike clade 2 and 3, which are mainly isolated from environmental sources [53]. Most of the isolates clustered in CC-828 (92.9%), in line with previously reported genotypes of clinical strains in Chile and the worldwide distribution of *C. coli* in birds and patients with diarrhea [53,80]. The only ST of *C. coli* that did not belong to any CC was the ST-1109, which has previously been isolated from animals and humans. These associations suggest that domestic broiler meat is likely an important source of antibiotic-resistant *Campylobacter* in Chile. Additionally, 21% (4/19) of the *C. jejuni* genomes did not match with previously reported STs, which correspond to four new STs (12300, 12301, 12302, 12303) found in this study.

The genotypes obtained in *A. butzleri* were only identified at ST level (genotypes did not cluster into clonal complexes), and corresponded to ST-40 and ST-172, which have only been described in samples associated with poultry (chicken, turkey, and poultry environment) in Nigeria, Thailand, and the USA. A total of 70% (7/10) of the genomes analyzed did not coincide with previously reported STs, corresponding to six potential new STs, which cannot be assigned yet due to the lack of a curator in the PubMLST database, as mentioned above. Due to the few studies carried out on this emerging pathogen and the late development of their MLST protocol compared to *Campylobacter* spp., knowledge of the epidemiology of *A. butzleri* is even more limited [81]. In fact, the data reported here correspond to the first MLST genotypes of *A. butzleri* in South America, which could provide a basis for understanding its distribution when data on clinical strains in the area are available.

## 4. Materials and Methods

### 4.1. Sample Collection

A total of 382 broiler meat packages were obtained from 14 supermarkets in the city of Valdivia (southern Chile), between June 2021 and January 2022. The meat came from the three main supply chains in Chile. This study was conducted by simple random sampling. The sample size was calculated using Working in Epidemiology “http://www.winepi.net/ (accessed on 10 March 2021)”, based on a seroprevalence of 46%, an error of 5%, and a 95% confidence interval [3]. The samples were transported refrigerated in an airtight bag to the laboratory, where they were analyzed within six hours.

### 4.2. Isolation of Campylobacterales

Three protocols were used for the isolation of different *Campylobacterales* genera.

Protocol A, aimed at the isolation of *Arcobacter*, consisted of enriching 10 g of meat in 90 mL of *Arcobacter* broth, supplemented with CAT (cefoperazone, amphotericin B and teicoplanin), homogenized for 1 min in a Stomacher bag and then incubated at 30 °C for 48 h in aerobic conditions. Then, 200 μL of broth was transferred to the surface of a Millipore membrane filter (diameter pore 0.45 μm) for passive filtration on Columbia Agar supplemented with 5% sheep blood, and allowed to filter for 30 min [82]. Finally, the filter was removed, and the plates were incubated at 30 °C for 48 h under aerobic conditions. Plates that were negative at 48 h were incubated for up to 5 days.

Protocol B, aimed at the isolation of *Campylobacter*, consisted of the enrichment of 10 g of meat in Bolton broth, supplemented with 5% sheep blood, homogenized for 1 min in a Stomacher bag and then incubated for 48 h at 37 °C under microaerobic condition using Anaerocult^©^ C (Merck Millipore). Next, 50 μL of this broth was streaked in mCCDA, which was incubated for 48 h at 37 °C under microaerobic conditions. Plates that were negative at 48 h were incubated for up to 5 days.

Protocol C1 and C2, aimed at the isolation of *Helicobacter*, consisted of adding 10 g of the sample to 90 mL of PBS 1X, and homogenizing it for 1 min in a Stomacher bag. This was then centrifuged, discarding the supernatant and resuspending the pellet of which 400 μL was used to carry out passive filtration as described above (Protocol C1). Another 50 μL was streaked in Columbia agar supplemented with 5% sheep blood and CAT antibiotic supplement. Both media were incubated under microaerobic conditions at 37 °C for 48 h (Protocol C2). Cultures that were negative at 48 h were incubated up to 7 days.

Suspicious colonies, with Gram characteristics of *Campylobacterales*, were cultured on Columbia agar supplemented with 5% blood sheep to obtain a pure culture. They were stored at −80 °C in BHI broth with 20% glycerol.

### 4.3. Identification

DNA extraction was performed from fresh bacterial culture using the boiling method [83]. Colonies obtained by protocols B and C were identified using multiplex PCR (mPCR) for *Campylobacter* [84]. Samples that resulted negative for the *Campylobacter* genus and isolates obtained from protocol A underwent PCR for *Arcobacter* genus, with the subsequent mPCR analysis for *Arcobacter* species [85,86].

### 4.4. Antimicrobial Susceptibility Testing

Antimicrobial susceptibility to CIP, E, TET, and GEN was assessed. The qualitative disk diffusion method was performed, for which the breakpoints of the Clinical and Laboratory Standards Institute (CLSI) M45 (2016) were used [87]. *Staphylococcus aureus* ATCC 25923 was used as a quality control strain. For GEN interpretation, the CLSI M100 (2020) guidelines for *Enterobacterales* were followed [88]. The minimal inhibitory concentration (MIC) was then determined using MIC Test Strip (Liofilchem) in the resistant isolates or in those with intermediate resistance.

### 4.5. Whole-Genome Sequencing

DNA of a set of representative antibiotic-resistant-strains (19 *C. jejuni*, 14 *C. coli* and 10 *A. butzleri*) were extracted using an Easy-DNA^TM^ gDNA Purification Kit (Invitrogen), and quantified by spectrophotometry (NanoQuant—Infinite M200, Tecan) and fluorometry (Qubit 3.0 fluorometer). The sample libraries were prepared using the Illumina DNA Prep kit and IDT 10 bp UDI indices, sequenced on an Illumina NextSeq 2000 at the Microbial Genomics Sequencing Center (SeqCenter, Pittsburgh, PA, USA). Sequence files were evaluated using FastQC v.0.11.9 before and after trimming [89]. Reads were trimmed using Trimmomatic v.0.39 to discard sequences with per base sequence quality score < 28 [90]. Two assemblers (SPAdes v3.13.1 and Unicycler v0.4.8) were used with default parameters and without reference-guided options. The assessment of draft genome assembly quality was done using the 3C criterion (contiguity, completeness, and correctness; Table S1) [91,92,93]. Assembly sequences were kept at the contig level with a minimum size of 1000 bp. The draft genomes were investigated for the presence of genes of antimicrobial resistance, which were identified using Comprehensive Antibiotic Resistance Database (CARD) and ResFinder [94]. Additionally, the sequences of the QRDR-*gyrA* gene from ciprofloxacin-resistant mutant strains in *A. butzleri* were analyzed in comparison with parental strains to identify point mutations. The detection of virulence determinants was carried out using the Virulence Factor Database (VFDB) and a local BLAST alignment (identified with > 90% identity and > 60% coverage). Genome assemblies were also mapped to PubMLST [95].

### 4.6. Molecular Detection of Helicobacter Pullorum

A subset of 50 samples, processed by protocol C, underwent DNA extraction using Cells and Tissue DNA Isolation Kit (Norgen Biotek, Thorold, ON, Canada), which were subjected to a specific PCR targeting to the *cdtB* gene of *Helicobacter pullorum* [96]. This was confirmed by Sanger sequencing and BLASTn analysis.

### 4.7. Phylogenomic Tree

The genome sequences from this study were uploaded to the Type (Strain) Genome Server (TYGS) (https://tygs.dsmz.de), for a whole genome-based taxonomic analysis. TYGS used the Genome-BLAST Distance Phylogeny method (GBDP) to compare whole-genome sequences. The phylogenomic tree was viewed and edited using iTOL v.4 [97].

### 4.8. Statistical Analysis

Prevalence among all *Campylobacterales* isolates in this study was assessed with Cochran’s Q test, while McNemar’s tests was used to determine any differences between the prevalence of *Campylobacter* and *Arcobacter* species. In both tests a *p*-value of <0.05 was considered significant. The analysis was performed using the statistical software R v4.1.1.

## 5. Conclusions

To our knowledge, this is the first study where three genera of the order *Campylobacterales* have been studied in parallel in samples of retail chicken meat using phenotypic and genomic approaches, showing a high prevalence and diversity of bacterial species along with high antibiotic resistance and virulence potential. In fact, emerging species such as *Arcobacter* spp. were identified with the highest prevalence. In addition, *Helicobacter pullorum* was reported for the first time in chicken meat in a South American country. Additionally, the concordance between the MLST genotypes of previously reported clinical strains of *Campylobacter* with those from chicken meat, as well as the presence of the same pattern of antibiotic resistance, leads us to suggest that poultry production in Chile bears part of the responsibility for the increase in antibiotic resistance observed in human campylobacteriosis. Therefore, epidemiological surveillance of *Campylobacterales* should be a priority for the Chilean food industry, and the governmental institutions should implement specific control measures to assess the contamination in chicken meat of national origin.

## Figures and Tables

**Table 1:** Prevalence and diversity of *Campylobacterales* isolated from 382 Chilean chicken meat samples.

Isolated Species	Positive Sample	
	N°	%
*C. jejuni*	43	11.3
*C. coli*	25	6.5
*A. butzleri*	96	25.1
*A. cryaerophilus*	7	1.8
*A. skirrowii*	4	1
*C. jejuni + C. coli*	2	0.5
*C. jejuni + A. butzleri*	27	7.1
*C. jejuni + A. cryaerophilus*	1	0.3
*C. coli + A. butzleri*	12	3.1
*C. coli + A. cryaerophilus*	1	0.3
*A. butzleri + A. cryaerophilus*	4	1
*A. butzleri + A. skirrowii*	1	0.3
*C. jejuni + C. coli + A. butzleri*	2	0.5
*C. coli + A. butzleri + A. cryaerophilus*	1	0.3
Total	226	59.2

**Table 2:** Prevalence of *Campylobacterales* using different isolation protocols.

	Isolation Protocols									
Species	A		B		C1		C2		Total (n = 382)	
	N°	%	N°	%	N°	%	N°	%	N°	% *
*C. jejuni*			75	19.6					75	19.6
*C. coli*			43	11.3					43	11.3
Total *Campylobacter*			114	29.8					114 **	29.8
*A. butzleri*	130	34	45	11.8			3	0.8	143	37.4
*A. cryaerophilus*	8	2.1	4	1	1	0.3	1	0.3	14	3.7
*A. skirrowii*	4	1					1	0.3	5	1.3
Total *Arcobacter*	142	37.2	49	12.8	1	0.3	5	1.3	156 **	40.8

A: Enrichment in *Arcobacter* broth supplemented with cefoperazone, amphotericin B and teicoplanin (CAT), incubated at 30 °C for 48 h under aerobic conditions and cultured by filtration over Columbia agar supplemented with 5% sheep blood (CBA), incubated at 30 °C under aerobic conditions for a minimum of 48 h. B: Enrichment in Bolton broth with Bolton antibiotic supplement, incubated at 37 °C under microaerobic condition for 48 h and cultured in modified charcoal cefoperazone deoxycholate agar (mCCDA) at 37 °C under microaerobic condition for a minimum of 48 h. C1: Suspension of sample in PBS, centrifugation, resuspension of the pellet which was filtrated over CBA and incubated at 37 °C under microaerobic conditions for a minimum of 48 h. C2: Suspension of sample in PBS, centrifugation, resuspension of the pellet which was cultured directly in CBA with supplement CAT and incubated at 37 °C under microaerobic conditions for 48 h. * Statistically significant difference was found among all prevalence of the isolated species (*p* < 0.05). ** More than one species was isolated from some samples.

**Table 3:** Distribution of antimicrobial resistance among *Campylobacterales* isolated from Chilean chicken meat.

Antimicrobial Agent	Species	n°	Disk Difussion Method				Test Strip																		
			Number of Strains				Distribution of MIC (µg/ml)																		
			S	I	R	%R	0.032	0.75	1.0	1.5	2	3	4	6	8	12	16	24	32	48	64	96	128	>256	%R
Ciprofloxacin	*C. jejuni*	75	47		28	37.3								1					27						37.3
	*C. coli*	43	19		24	55.8								1					23						55.8
	*A. butzleri*	143	135		8	5.6	1				3		1	1	1				1						2.8
	*A. cryaerophilus*	15	15																						
	*A. skirrowii*	4	4																						
Erythromycin	*C. jejuni*	75	75																						
	*C. coli*	43	36		7	16.3																		7	16.3
	*A. butzleri*	143	140	2	1	1									1			1	1						0.7
	*A. cryaerophilus*	15	15																						
	*A. skirrowii*	4	4																						
Tetracycline	*C. jejuni*	75	60		15	20											1		5	5	2	1	1		20
	*C. coli*	43	41		2	4.7													1	1					4.7
	*A. butzleri*	143	59	32	52	36.4			1	1	16	9	22	14	11	6	4								2.8
	*A. cryaerophilus*	15	12	2	1	6.7				1	1	1													
	*A. skirrowii*	4	2	2							2														
Gentamicin	*C. jejuni*	75	75																						
	*C. coli*	43	43																						
	*A. butzleri*	143	143																						
	*A. cryaerophilus*	15	15																						
	*A. skirrowii*	4	4																						

n°, number of isolates. S, susceptible; I, intermediate; R, resistant; %R, percentage of resistance. Minimum Inhibitory Concentration (MIC) breakpoints: ciprofloxacin ≥ 4 mg/L, erythromycin ≥ 32 mg/L, gentamicin ≥ 16 mg/L, tetracycline ≥ 16 mg/L. The grey shading indicates resistant isolates.

## Data Availability

The datasets presented in this study can be found in NCBI under Bioproject PRJNA932782.

## Supplementary Materials

The following supporting information can be downloaded at: https://www.mdpi.com/article/10.3390/antibiotics12050917/s1, Table S1. Comparison of contiguity and annotation of genomes assemblies by different approaches.; Figure S1. Phylogenetic tree based on whole-genome sequencing generated with Genome Blast Distance Phylogeny (GBDP). The presence or lack of virulence genes in antibiotic-resistant *Campylobacter* spp. is indicated by the presence or absence of red blocks, respectively. The new STs are in bold type. Bar, 0.01.; Figure S2. Phylogenetic tree based on whole-genome sequencing generated with Genome Blast Distance Phylogeny (GBDP). The presence or lack of virulence of antibiotic-resistant *Arcobacter butzleri* is indicated by the presence or absence of red block, respectively. NST: New Sequence Type. NST are in bold type. Bar, 0.01.; Figure S3. Phylogenetic tree based on whole-genome sequencing generated with Genome Blast Distance Phylogeny (GBDP). This shows the clade to which the *C. coli* strains isolated in this study belong. RM2228 belongs to clade 1, H055260513 to clade 2, and BIGS0008 to clade 3. Bar, 0.01.
