# Supplementary material for: Genomic Characterization of Antibiotic-Resistant Campylobacterales Isolated from Chilean Poultry Meat
Source: Antibiotics (Basel). 2023 May 16;12(5):917. doi: 10.3390/antibiotics12050917 (PMC10215856; doi:10.3390/antibiotics12050917)
Supplement: Supplementary file 1 [file antibiotics-12-00917-s001.zip › Figure S1.pdf]

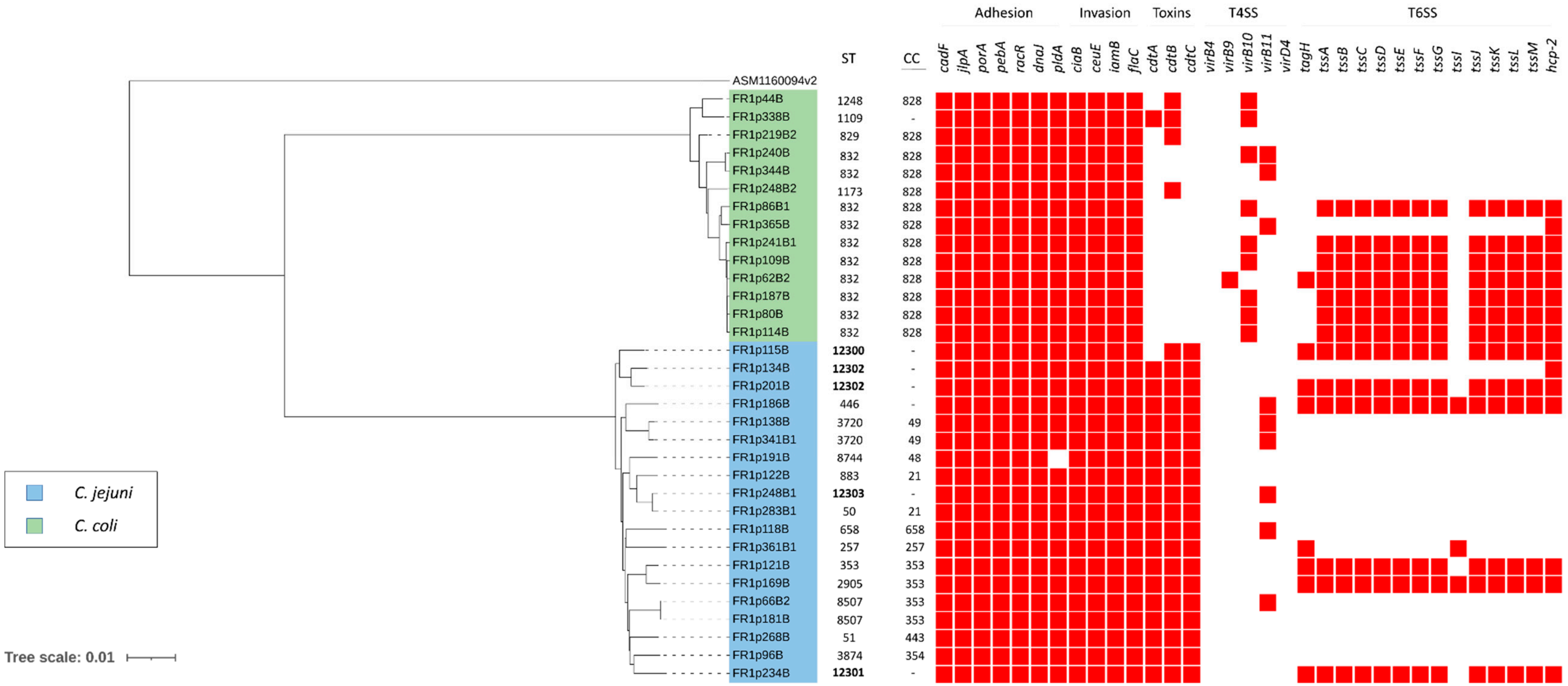

Figure S1. Phylogenetic tree based on whole-genome sequencing generated with Genome Blast Distance Phylogeny (GBDP). The presence or lack of virulence genes in antibiotic-resistant *Campylobacter* spp. is indicated by the presence or absence of red block, respectively. The new ST are in bold type. Bar, 0.01
