# Supplementary material for: Genomic Characterization of Antibiotic-Resistant Campylobacterales Isolated from Chilean Poultry Meat
Source: Antibiotics (Basel). 2023 May 16;12(5):917. doi: 10.3390/antibiotics12050917 (PMC10215856; doi:10.3390/antibiotics12050917)
Supplement: Supplementary file 1 [file antibiotics-12-00917-s001.zip › Figure S2.pdf]

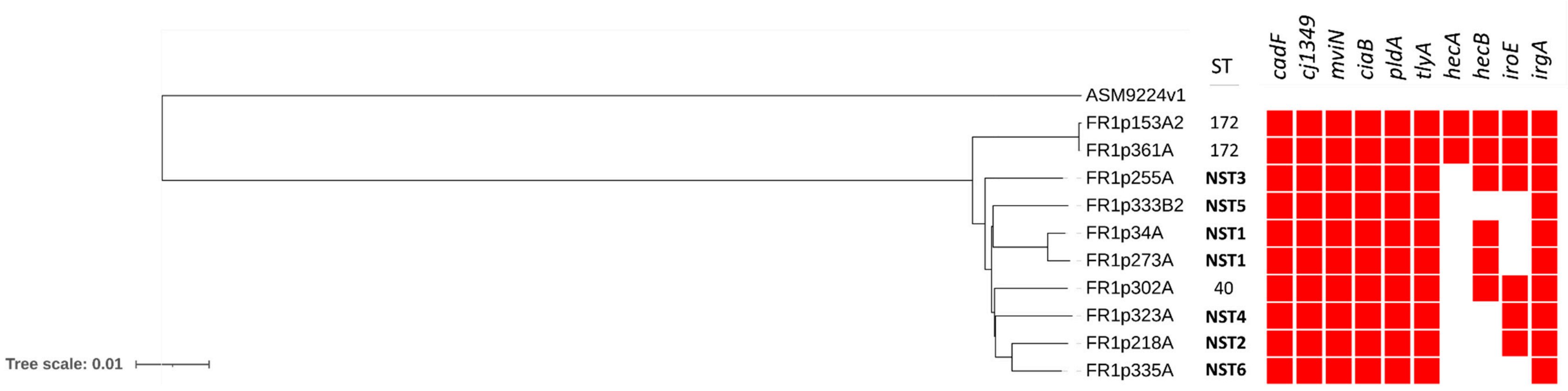

Figure S2**.** Phylogenetic tree based on whole-genome sequencing generated with Genome Blast Distance Phylogeny (GBDP). The presence or lack of virulence of antibiotic-resistant *Arcobacter butzleri* is indicated by the presence or absence of red block, respectively. NST: New Sequence Type. NST are in bold type. Bar, 0.01.
