# Supplementary material for: Genomic Characterization of Antibiotic-Resistant Campylobacterales Isolated from Chilean Poultry Meat
Source: Antibiotics (Basel). 2023 May 16;12(5):917. doi: 10.3390/antibiotics12050917 (PMC10215856; doi:10.3390/antibiotics12050917)
Supplement: Supplementary file 1 [file antibiotics-12-00917-s001.zip › Figure S3.pdf]

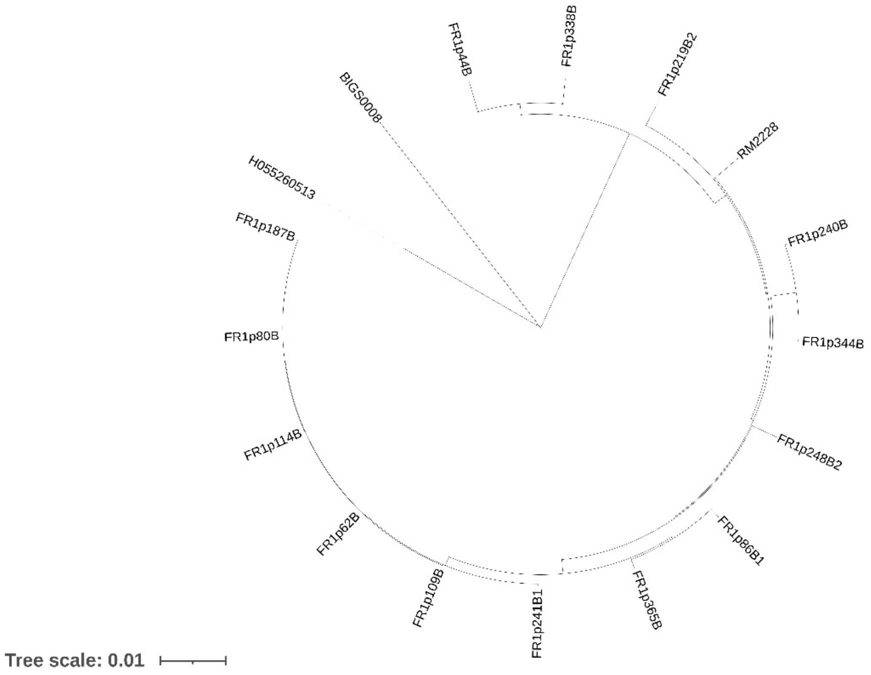

Figure S3. Phylogenetic tree based on whole-genome sequencing generated with Genome Blast Distance Phylogeny (GBDP). This shows the clade to which the *C. coli* strains isolated in this study belong. RM2228 belongs to clade 1, H055260513 to clade 2 and BIGS0008 to clade 3. Bar, 0.01
